# Results from a large post-marketing safety surveillance study in the Republic of Korea with a quadrivalent meningococcal CRM-conjugate vaccine in individuals aged 2 months–55 years

**DOI:** 10.1080/21645515.2019.1670125

**Published:** 2019-10-25

**Authors:** Byung Wook Yoo, Hye Lim Jung, Yoon Seob Byeon, Dong Ki Han, Nak Yeong Jeong, Carlo Curina, Luca Moraschini, Sung Jin Kim, Chiranjiwi Bhusal, Michele Pellegrini, Yan Miao

**Affiliations:** a Department of Family Medicine, Soonchunhyang University Seoul Hospital, Seoul, Republic of Korea; b Department of Pediatrics, Sungkyunkwan University, Kangbuk Samsung Hospital, Seoul, Seoul, Republic of Korea; c Department of Pediatrics, Moran Women’s Hospital, Gyeongsangnam-do, Republic of Korea; d Pediatrics Clinic, Gyeongsangnam-do, Republic of Korea; e Department of Internal Medicine, Yonsei Koum Internal Medicine Clinic, Seoul, Republic of Korea; f GSK, Siena, Italy; g GSK, Seoul, Republic of Korea; h GSK, Amsterdam, The Netherlands

**Keywords:** Safety surveillance, MenACWY-CRM, children, adolescents, adults, Republic of Korea

## Abstract

The quadrivalent meningococcal conjugate vaccine MenACWY-CRM is approved in the Republic of Korea for use in individuals from 2 months of age. This single-arm, open-label, observational, multicenter, post-marketing study (NCT01766206) assessed the safety of MenACWY-CRM vaccine administered according to local clinical practice. A total of 3939 individuals aged 2 months–55 years provided safety data post-vaccination; the analysis was conducted on the per-protocol set (3920 participants). Solicited and unsolicited adverse events (AEs) were collected over 7 days post-vaccination and medically-attended AEs (MAAEs) and serious AEs (SAEs) over 29 days post-vaccination. Among recorded solicited AEs, injection site AEs were reported by 21.38% of participants, with tenderness/pain being most frequent across age groups; systemic AEs were reported in 13.95% of participants, with irritability (in ˂6-year-olds), headache and myalgia (in ≥6 year-olds) being the most frequently reported. Most solicited AEs were mild or moderate in nature. The percentage of participants reporting unsolicited AEs varied in the study population, i.e. 12.56% in participants aged 2–23 months and 3.18% in those ≥2 years of age. Overall, less than 22% of unsolicited AEs were considered as related to vaccination. MAAEs (10.89% of participants) were mostly mild; 2.82% were considered as related to vaccination. Three (0.46%) and 5 (0.15%) SAEs (none vaccination-related) occurred in participants aged 2–23 months and 2–55 years, respectively. No deaths were reported. The safety profile for MenACWY-CRM in this post-marketing surveillance was consistent with observations from studies conducted during the vaccine’s clinical development, with no new safety concerns.

## Introduction

Invasive meningococcal disease (IMD) is an acute bacterial infection caused by *Neisseria meningitidis*, with six meningococcal serogroups (A, B, C, W, Y and more recently, X) accounting virtually for all IMD cases worldwide.^1,2^ Although a decrease in the incidence of the disease has been noted in the last decade, IMD remains a significant global public health threat: the case fatality ratio is around 10% with treatment, and long-term sequelae are observed in up to 20% of survivors.^3,4^ Young people are at the highest risk of contracting the disease, with incidence peaks observed in infants, and to a lesser extent in adolescents and young adults.^2^

The epidemiology of IMD is dynamic, with great variations in the prevalence of disease-causing serogroups observed from one region to another, and over time.^1,2,5–7^ In the Republic of Korea, the incidence of meningococcal disease is relatively low according to the national surveillance system, with annual estimates of 0.01–0.08 cases per 100,000 persons, although these reports are believed to underestimate the true burden of the disease.^7,8^ According to the Korea Centers for Disease Control and Prevention data, the annual number of meningococcal meningitis cases was 5–6 between 2013 and 2016, but increased to 17 in 2017 and was estimated at 12 in August 2018.^9^ Between 2010 and 2016, serogroup B was the most prominent cause of IMD in the Republic of Korea identified in 37% of cases, followed by serogroups W (26%) and C (21%).^10^

Vaccination remains the most effective approach to prevent IMD.^2^ Polysaccharide meningococcal vaccines were initially used, but were observed to not be immunogenic in children and unable to elicit immunological memory; the focus therefore shifted toward the development of protein-conjugate formulations. Mass vaccination with the monovalent serogroup C conjugate meningococcal vaccine, first implemented by the United Kingdom, was successful, with a rapid decline in the incidence of serogroup C IMD in vaccinated and unvaccinated populations.^1^ Subsequently, quadrivalent meningococcal vaccines, targeting serogroups A, C, W and Y, have been developed and licensed worldwide. The meningococcal ACWY quadrivalent CRM_197_-conjugate vaccine (MenACWY-CRM; *Menveo*, GSK) is approved for use in different age groups in many countries.^11^ The vaccine has been shown to be immunogenic, able to prime immunological memory, and well tolerated in infants,^12,13^ children,^14^ adolescents and adults.^15–17^

MenACWY-CRM was initially licensed in the Republic of Korea for use in individuals aged 11–55 years in 2012, and subsequently in children aged 2–11 years and infants ≥2 months of age in 2013 and 2014, respectively. MenACWY-CRM is the first meningococcal vaccine approved for use in infants in this country. This surveillance study was conducted as a post-marketing commitment to the Ministry of Food and Drug Safety (MFDS) of the Republic of Korea, to monitor the safety of marketed MenACWY-CRM in participants aged 2 months–55 years receiving the vaccine according to routine clinical practice and prescribing information.

**Figure 1.**
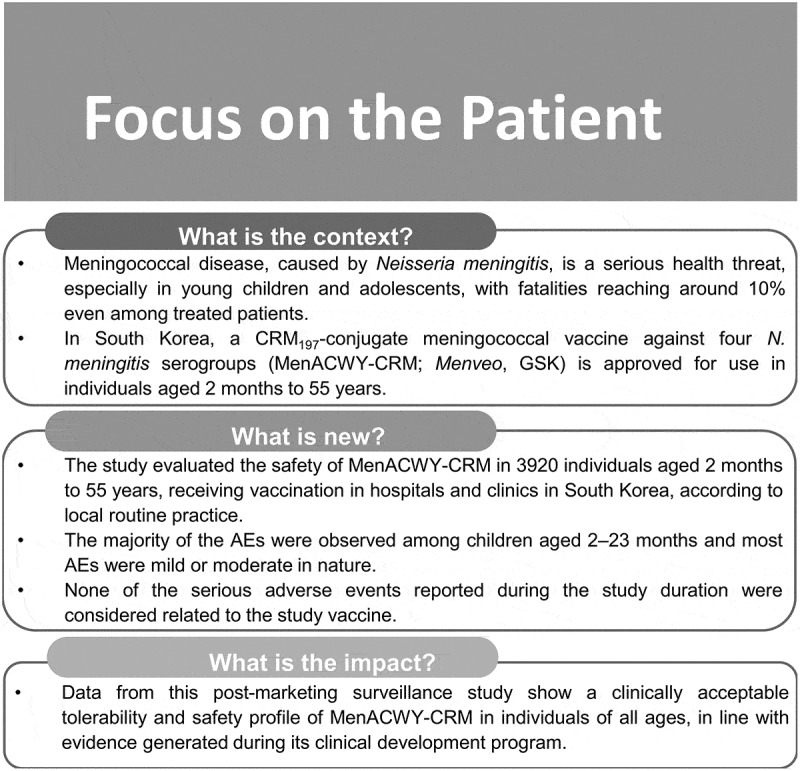
Focus on the patient section.

The Focus on the Patient section (Figure 1) summarizes the present research and its clinical relevance and impact on the vaccinated population.

## Methods

### Study design and participants

This single-arm, open-label, multi-center, observational, post-marketing surveillance study was conducted between May 2012 and May 2018 in the Republic of Korea, and was designed to monitor the safety of MenACWY-CRM administered according to routine clinical practice in healthy participants aged 2 months–55 years.

Safety data were collected for a 29-day period (day 1–day 29) following the receipt of a MenACWY-CRM dose which was administered according to local prescribing information in each age category: as a 4-dose series in children initiating vaccination at 2–6 months of age (three doses administered at least two months apart, with the fourth dose in the second year of life), as a 2-dose series in unvaccinated children 7–23 months of age (with the second dose administered in the second year of life, at least three months after the first dose), or as a single dose in participants aged 2–55 years. Children 2–23 months of age were enrolled at any point in the vaccination series and were followed-up after each dose of MenACWY-CRM received, according to parental consent.

Individuals were considered eligible for enrollment if: (i) they were 2 months–55 years of age at the time of MenACWY-CRM administration, (ii) written informed consent/assent was obtained by them or their parent/legal representative, (iii) they were able to comply with the protocol’s requirements (e.g. completion of the diary card), and (iv) they were in good health as determined by their medical history outcome and the physical assessment and clinical judgment of the investigator. Concomitant routine vaccinations according to the national recommendations in South Korea^18^ were allowed. Individuals were excluded if: (i) they had a contraindication, special warnings and/or precautions reported in the MenACWY-CRM prescribing information as evaluated by the investigator, and (ii) they had already been enrolled in the study at another study site for previous MenACWY-CRM vaccination.

A 0.5 mL dose of MenACWY-CRM contains 10 μg of *N. meningitidis* serogroup A oligosaccharide and 5 μg each of oligosaccharides from serogroups C, W and Y, conjugated to CRM_197._The vaccine was reconstituted immediately before administration by mixing the lyophilized A component with the liquid CWY component and administered by intramuscular injection, preferably in the anterolateral aspect of the thigh in infants or into the deltoid muscle (upper arm) in children, adolescents and adults.^11^

This study was conducted in accordance with International Council for Harmonized Tripartite Guidelines for Good Clinical Practice, the MFDS requirements and regulations, and the Declaration of Helsinki. The study was registered at www.clinicaltrials.gov (NCT01766206) and a protocol summary is available from www.gsk-clinicalstudyregister.com (study ID 205341).

### Study objectives and endpoints

The primary objective of the study was to monitor the safety of MenACWY-CRM in individuals aged 2 months to 55 years in the Republic of Korea, as assessed in terms of number of participants exposed to the study vaccine with reported: solicited local and systemic adverse events (AEs) and unsolicited AEs within the 7-day period (day 1–7) post-vaccination, and medically-attended AEs (MAAEs) and serious AEs (SAEs) within the 29-day period post-vaccination.

Solicited local AEs were injection site erythema, injection site induration and injection site tenderness (children <6 years of age) or pain (participants aged ≥6 years). Solicited systemic AEs were changes in eating habits, sleepiness, irritability, rash, vomiting, diarrhea and fever for children aged <6 years, and chills, nausea, malaise, generalized myalgia, generalized arthralgia, headache, rash and fever for those ≥6 years of age (Supplementary Material, Table S1).

MAAEs were defined as events requiring a physician’s or an emergency room visit.

All AEs were graded by severity, from mild to severe, as shown in Table S1.

All AEs/SAEs were assessed by the investigator as either probably related, possibly related, or unrelated to MenACWY-CRM vaccination. Any AEs resulting in withdrawal from the study were also summarized. Unsolicited AEs were recorded by preferred terms using the most recent Medical Dictionary for Regulatory Activities and grouped by system organ class.

### Statistical analysis

A total of 3960 participants were planned for enrollment in this study (assuming a 10% drop-out rate) to obtain approximately 3000 evaluable participants in the 2–55 years of age cohort, and 600 participants in the 2–23 months of age cohort, to provide continued safety monitoring in the Korean population in compliance with post-licensure safety MFDS requirements. All planned analyzes were purely descriptive and were initially conducted by age strata (2–10 years, 11–18 years, 19–34 years and 35–55 years of age). Following a request from the MFDS, post-hoc analyses were performed to present safety data separately for the 2–23 months and 2–55 years age groups.

## Results

### Demographics

**Figure 2.**
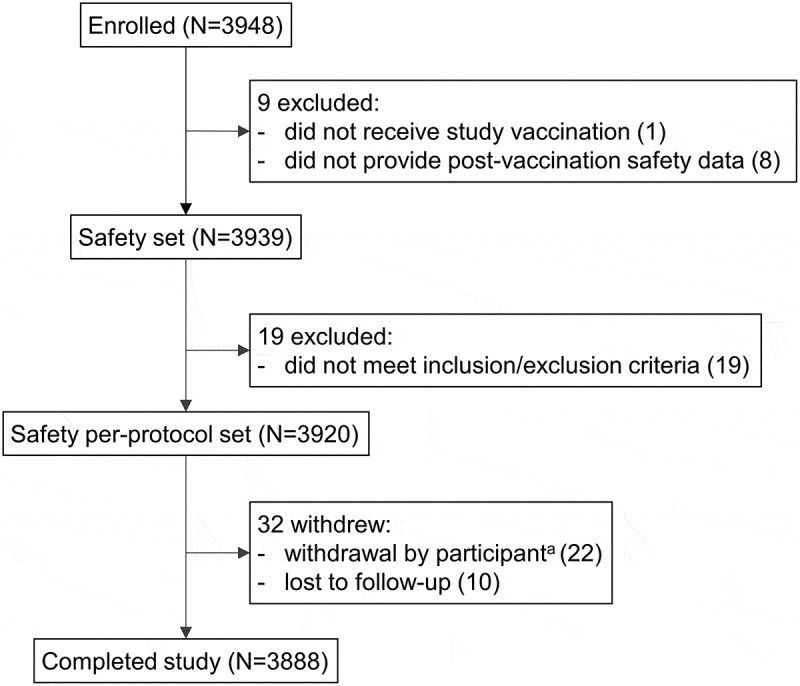
Participant flow chart.

A total of 3948 individuals were enrolled in the study: 3939 provided safety data and 3920 were included in the safety per-protocol set (with no protocol deviations). Failure to meet inclusion/exclusion criteria was the main reason for exclusion from the per-protocol set (Figure 2). The demographic characteristics of participants at enrollment are presented in Table 1. The mean age in the safety per-protocol set was 18.21 years and 83.34% of participants belonged to the 2–55 years age group. There were slightly more female (52.53% in the 2–23 months and 56.01% in the 2–55 years age groups, respectively) than male participants. Most participants in either age group received 1 dose of MenACWY-CRM (Table 1).

**Table 1.** Baseline characteristics of study participants (per protocol safety set; N = 3920).

	2–23 months	2–55 years
Age (mean ± standard deviation)	8.59 ± 5.74	21.78 ± 14.16
Age, n (%)	653 (16.66)	3267 (83.34)
2–5 years	-	551 (14.06)
6–10 years	-	338 (8.62)
11–18 years	-	431 (10.99)
19–34 years	-	1286 (32.81)
35–55 years		661 (16.86)
Female, n (%)	343 (52.53)	1830 (56.01)
Ethnic origin, n (%)		
Asian	652 (99.85)	3266 (99.97)
Caucasian	0 (0.0)	1 (0.03)
Hispanic	1 (0.15)	0 (0.0)
MenACWY-CRM doses received, n (%)		
1	304 (46.55)	3256 (99.66)
2	140 (21.44)	5 (0.15)
3	99 (15.16)	0 (0.00)
4	110 (16.85)	6 (0.18)

N, number of participants; n (%), number (percentage) of participants in each category.

### Safety data

During the surveillance period, a total of 3011 AEs; including solicited, unsolicited and SAEs; were reported in 1376 study participants, with 35.10% of participants experiencing at least one AE (Table 2). The percentage of participants with at least one reported AE was higher in the 2–23 months age group (64.47%), compared to the 2–55 years one (29.23%) (Table 2). Overall, no increase in the reporting of AEs was observed with subsequent vaccinations in the 2–23 months age group (Table S2).

**Table 2.** Summary of reported adverse events, overall and by age group (safety per protocol set).

Type	n (%)	Number of AEs
Total AEs	1376 (35.10)	3011
2–23 months	421 (64.47)	1185
2–55 years	955 (29.23)	1826
Solicited AEs	1139 (29.06)	2262
2–23 months	321 (49.16)	807
2–55 years	818 (25.04)	1455
Unsolicited AEs	186 (4.74)	268
2–23 months	82 (12.56)	111
2–55 years	104 (3.18)	157
SAEs	8 (0.20)	8
2–23 months	3 (0.46)	3
2–55 years	5 (0.15)	5
MAAEs ^a^	427 (10.89)	674
2–23 months	238 (36.45)	361
2–55 years	189 (5.79)	313

AE, adverse event; n (%), number (percentage) of participants with at least one AE; SAE, serious AE; MAEE, medically-attended AE.

Note: ^a^193 cases of MAAEs were also included in solicited systemic (5 cases) and unsolicited (188 cases) AEs.

## Solicited adverse events

**Figure 3.**
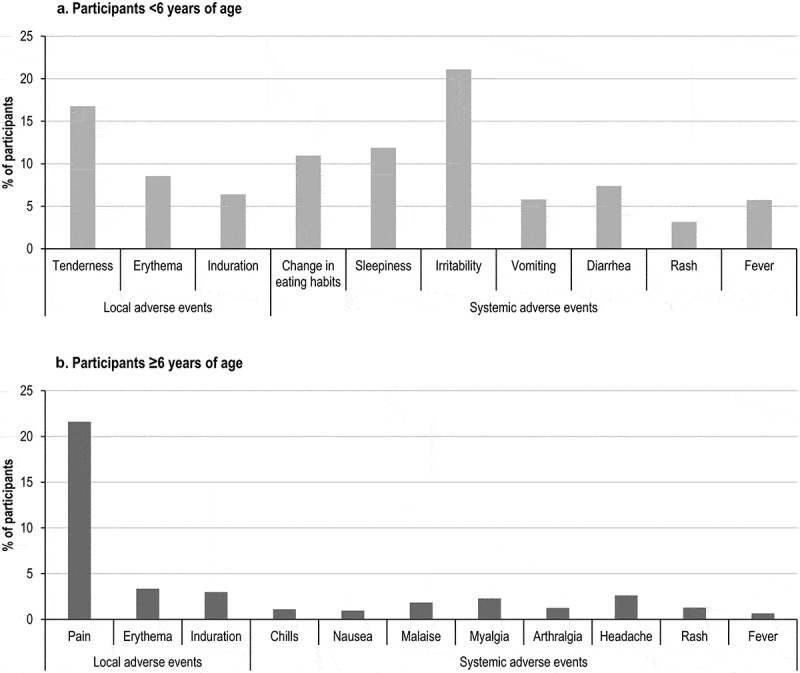
Occurrence of solicited local and systemic adverse events, by age group (safety per protocol set).

The most frequently reported solicited local AEs were injection site tenderness (in 16.78% of participants <6 years of age) and pain (in 21.61% of participants aged ≥6 years) (Figure 3(a,b)). The most frequently reported solicited systemic AEs were irritability (in 21.10% of children aged <6 years), and headache and myalgia (in 2.61% and 2.28% of participants aged ≥6 years of age, respectively) (Figure 3(a,b)).

**Figure 4.**
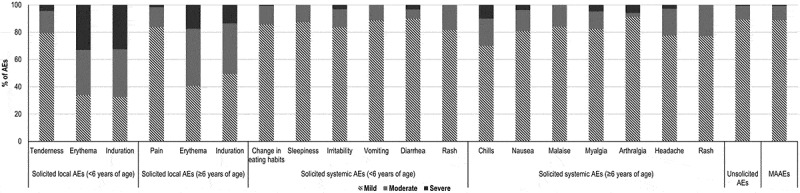
Distribution of solicited, unsolicited and medically-attended adverse events by severity (in participants aged ˂6 years and ≥6 years).

Solicited AEs were generally mild or moderate in nature. Among solicited local AEs, 4.46–33.01% in children <6 years and 1.70–17.58% in participants ≥6 years were severe. The proportion of severe events among solicited systemic AEs ranged from 0.00%–3.37% and from 0.00%–10.00% in participants <6 years and ≥6 years of age, respectively (Figure 4).

## Unsolicited adverse events

Overall, unsolicited AEs were reported in 4.74% of participants. Higher percentages of participants with reported unsolicited AEs were observed for the 2–23 months age group (12.56%), compared to the 2–55 year age group (3.18%) (Table 2).

In the 2–23 month age group, the most frequently unsolicited AEs were nasopharyngitis (in 3.22% of participants), bronchitis (in 2.14% of participants), pharyngitis and bronchiolitis (each in 1.07% of children). In the 2–55-year-olds, the most frequent AEs were bronchitis (in 1.04% of participants), nasopharyngitis (in 0.70% of participants), pharyngitis (0.64% of participants) and gastroenteritis (0.55% of participants).

Most unsolicited AEs were mild (89.18%) or moderate (10.07%) in nature, with only 0.75% reported as severe (Figure 4). In total, 21.65% of the unsolicited AEs (occurring in 47 [1.20%] participants of all ages) were considered as possibly or probably related to the study vaccine.

## Medically-attended adverse events

In total, 674 MAAEs occurred in 427 (10.89%) participants in the safety per-protocol set within the 29-day post-vaccination period. MAAEs were reported with a higher frequency in children aged 2–23 months (36.45%), compared to participants aged 2–55 years (5.79%) (Table 2). In the 2–23 months age group, nasopharyngitis, bronchitis, and pharyngitis were the most frequent MAAEs, reported in 9.49%, 9.34%, and 3.22% of children, respectively. The same MAAEs were also the most frequently reported in the 2–55 years age group, but in a much lower percentage of participants (in 2.26% for nasopharyngitis, 4.08% for bronchitis and 2.25% for pharyngitis) compared to the younger age group.

The majority of MAAEs (88.72%) were considered mild in nature, and only 0.89% were classified as severe (Figure 4). By study end, 80.56% of MAAEs were resolved and 19.29% persisted beyond the 29-day post-vaccination period, with 0.15% (1) lost to follow-up. Overall, 2.82% of the MAAEs (occurring in 0.46% [18] of participants) were considered as possibly or probably related to the study vaccine.

## Serious adverse events

Within the 29-day post-vaccination period, a total of 8 SAEs were reported (Table 2). Three SAEs (bronchiolitis, pneumonia and tonsillitis) were reported in the 2–23 months age group, and 5 were reported in participants aged 2–55 years (two cases of pneumonia and one of pyrexia in the 2–10 years age stratum, and a case of pneumonia and one of dizziness in the 11–18 years age stratum). None of the SAEs were considered related to the study vaccine. No fatal events were reported.

The results of additional analyses by age strata (including the 11–55 yeas age group) are presented in Table S3.

## Discussion

This is the first study assessing the safety of MenACWY-CRM administered according to routine local immunization practices in the Republic of Korea. Overall, 3948 participants aged 2 months – 55 years were enrolled, and 3920 were followed up for approximately one month after MenACWY-CRM vaccination and included in the study analysis. Overall, AEs were reported in 35.10% of study participants. The majority of the AEs were observed among children aged 2–23 months and most AEs were mild or moderate in nature. Eight SAEs were reported during the study but none were considered as related to the study vaccine.

The safety data observed in this post-marketing surveillance, observational study is consistent with the current safety profile of MenACWY-CRM as observed during its clinical development program.^19^ Overall, a slightly lower occurrence of AEs was observed in this study conducted in the Republic of Korea, when compared with safety data collected in other geographical areas, in line with what was previously noted in pivotal clinical trials in Asian countries, such as South Korea,^20^ Taiwan^21^ and India.^22^

As already shown in other clinical trials in which MenACWY-CRM was administered alone or with routine pediatric vaccines, injection site tenderness and irritability were the most frequently reported solicited local and systemic AEs for young children<2 years of age.^12,13,23–25^ Overall, in pre-licensure trials, solicited AEs were observed in ≥50% of children receiving vaccination until 23 months of age, in line with observations made in our study. Unsolicited AEs occurred with similar or lower frequency to that reported in a large phase 3 multi-country pivotal safety study in 5772 infants, who received MenACWY-CRM co-administered with routine vaccines, with overall incidences of AEs observed up to 84%.^12^ A relatively lower frequency of SAEs (0.6%) was observed in our study compared with data from pre-licensure trials, in which rates of up to 3.6% were reported in infants and toddlers receiving MenACWY-CRM at 2, 4, 6 and 12 months of age.^11^ This frequency was also lower than that observed in the pivotal safety study, where SAEs were reported in 6% of participants aged below 2 years.^12^ Of note, in these clinical trials, SAEs were collected over the entire study (from first vaccination to approximately six months post last vaccination), which constitutes a longer follow-up interval than the 29-day period post each vaccination in our study.

As expected, the incidence of reported AEs was lower in the 2–55 year-olds than in children <2 years of age during this post-marketing surveillance. Moreover, this incidence was similar or even lower than that emerging from clinical studies in children, adolescents and adults in this age group, for whom an acceptable clinical safety profile was shown.^11^ Safety data are available for children 2–10 years of age from various trials conducted in Europe, the United States (US), Latin America^13,17,22^ and Asia.^21,22^ Studies in adolescents and adults aged 11–55 years on MenACWY-CRM administered alone or with other vaccines (tetanus-diphtheria-acellular pertussis or human papillomavirus vaccines) are also available from the US,^17,26^ Italy^27^ and Latin America (Argentina, Colombia^28^ and Costa Rica^15^).

Consistent with observations in our study, in a pre-licensure trial conducted in the United States and Canada in children aged 2–5 years, injection site tenderness/pain (in 28–33% of participants) was the most frequently reported solicited local AE, while irritability (in 16–22% of children) and sleepiness (12–17%) were the most frequent systemic AEs following a MenACWY-CRM vaccination.^14^ Severe reactions were rarely reported.^14^ Results from our study were also consistent with previous reports in individuals aged 6 years and above: pain (in 13.3–39%) was the most commonly reported solicited local AEs, while headache, malaise and myalgia were the most frequent systemic AEs for children aged 6–10 years in trials conducted in the US, Canada^14^ and Asia.^21,22^ In adolescents and adults, pain (for ≤54% of participants), headache and myalgia (for ≤41% of participants) were the most common local and systemic AEs, respectively, and very few severe reactions occurred.^15,17,20,26–28^

Following MenACWY-CRM vaccination in pre-licensure studies, unsolicited AEs were observed in 19%^28^ to 42%^26^ of participants aged 2–55 years, including in children aged 2–10 years (up to 26%).^14^ A lower incidence of unsolicited AEs (in <3.2% of participants) was observed in the current surveillance. Similarly, SAEs were reported in up to 0.7% of participants 2–55 years of age within a 6–12 months follow-up period in previous trials,^11^ compared to 0.15% within 29-day period post-vaccination in our study. When compared with results from a previous clinical study in adolescents and adults aged 11–55 years in South Korea,^20^ a lower incidence of solicited local (20.65% versus 28%) and systemic AEs (6.01% versus 28%) was observed in the current surveillance for the same age group. Moreover, a substantially lower incidence of unsolicited AEs (1.26% versus 12%) was reported in participants aged 11–55 years in this post-marketing surveillance compared to the clinical trial conducted in South Korea.^20^

The current study results are also in line with those from post-licensure surveillance studies in the US, based on data from the Vaccine Adverse Event Reporting System (VAERS) available for a 5-year period in all age groups.^29^ Most of the US reports emerged from the 11–18 years age group as routine meningococcal vaccination is recommended in adolescents; the most frequently reported AEs were related to local reactions, dizziness, pyrexia and headache. Among the 2614 US reports received following MenACWY-CRM vaccination, 67 were identified as serious, with anaphylaxis and syncope being the most frequent. However, in line with the present Republic of Korean study, VAERS data were aligned with the already known pre-licensure safety data, and no new safety concerns were identified for MenACWY-CRM.^29^

The observational design of this post-marketing safety surveillance study, including the administration of the vaccine according to field practice – therefore allowing for concomitant vaccination – represents a potential limitation. However, the safety profile of MenACWY-CRM in the routine clinical settings of our study is consistent with other studies conducted in the Asian population,^18–20^ including one study conducted in 450 Korean adolescents and adults aged 11–55 years.^20^ In addition, although the study has been carried out in full compliance with the national guidelines on post-marketing safety surveillance, the size of the study may not allow the capture of very rare or uncommon AEs, and there was no control group.

## Conclusion

Data from this post-marketing surveillance study in participants aged 2 months to 55 years showed a clinically acceptable tolerability and safety profile of MenACWY-CRM, in line with the evidence generated across its clinical development program.

## Supplementary Material

Supplemental Material

## Abbreviation list

AE: Adverse event
IMD: Invasive meningococcal disease
MAAE: Medically-attended adverse event
MenACWY-CRM: Meningococcal serogroup A, C, W and Y quadrivalent CRM_197_-conjugate vaccine
MFDS: Ministry of Food and Drug Safety
SAE: Serious adverse event
US: United States
VAERS: Vaccine Adverse Event Reporting System.

## Data sharing statement

The results summary for this study (GSK study number 205341 – NCT01766206) is available on the GSK Clinical Study Register and can be accessed at www.gsk-clinicalstudyregister.com. For interventional studies that evaluate our medicines, anonymized patient-level data will be made available to independent researchers, subject to review by an independent panel, at www.clinicalstudydatarequest.com within six months of publication. To protect the privacy of patients and individuals involved in our studies, GSK does not publicly disclose patient-level data.

## Trademark

*Menveo* is a trademark owned by the GSK group of companies.
