# Supplementary material for: Results from a large post-marketing safety surveillance study in the Republic of Korea with a quadrivalent meningococcal CRM-conjugate vaccine in individuals aged 2 months–55 years
Source: Hum Vaccin Immunother. 2019 Oct 25;16(6):1260–7. doi: 10.1080/21645515.2019.1670125 (PMC7482729; doi:10.1080/21645515.2019.1670125)
Supplement: Supplemental Material [file KHVI_A_1670125_SM1774.zip › Table S1.docx]

# Table S1. Grading of severity for adverse events

|  |  | Grading of severity | | | |  |
| --- | --- | --- | --- | --- | --- | --- |
|  |  | Mild | Moderate | Severe |  |  |
| Children aged <6 years | | | | | | |
| Solicited  local AEs | Injection site tenderness | Minor light reaction to touch | Cried or protested to touch | Cried when injected limb was moved |  |  |
|  | Injection site erythema and induration | 10–25 mm | 26–50 mm | >50 mm |  |  |
| Solicited  systemic AEs* | Change in eating habits | Eating less than normal for 1 or 2 feeds | Missed 1 to 2 feeds | Missed more than 2 feeds |  |  |
|  | Sleepiness | Shows an increased drowsiness | Sleeps through feeds | Sleeps most of the time and it is hard to arouse him/her |  |  |
|  | Irritability | Requires more cuddling, less playful than usual | More difficult to settle | Unable to console |  |  |
|  | Vomiting | 1 or 2 episodes/24 hours | >2 episodes/24 hours | Requires outpatient hydration |  |  |
|  | Diarrhea | 2–3 loose stools or <400 gms/24 hours | 4-5 stools or 400–800 gms/24 hours | 6 or more watery stools or >800gms/24 hours or requires outpatient IV hydration |  |  |
| Unsolicited AEs | | Transient with no limitation in normal daily activity | Some limitation in normal daily activity | Unable to perform normal daily activity |  |  |
| Individuals aged ≥6 years | | | | | | |
| Solicited  local AEs | Injection site pain | Present but does not interfere with activity | Interferes with activity | Prevents daily activity |  |  |
|  | Injection site erythema and induration | 25–50 mm | 51–100 mm | >100 mm |  |  |
| Solicited  systemic AEs* |  | Present but does not interfere with activity | Interferes with activity | Prevents daily activity |  |  |
| Unsolicited AEs |  | Transient with no limitation in normal daily activity | Some limitation in normal daily activity | Unable to perform normal daily activity |  |  |

AE, adverse event.

Note: * For all age categories, rash was classified as none, urticarial or other and body temperature as <38℃ (no fever) or ≥38℃ (fever).
