# Supplementary material for: Results from a large post-marketing safety surveillance study in the Republic of Korea with a quadrivalent meningococcal CRM-conjugate vaccine in individuals aged 2 months–55 years
Source: Hum Vaccin Immunother. 2019 Oct 25;16(6):1260–7. doi: 10.1080/21645515.2019.1670125 (PMC7482729; doi:10.1080/21645515.2019.1670125)
Supplement: Supplemental Material [file KHVI_A_1670125_SM1774.zip › Table S3.docx]

# Table S3. Summary of reported adverse events, overall and by age group (safety per protocol set)

|  | n (%) | Number of AEs |
| --- | --- | --- |
| Total AEs | 1139 (29.06) | 2262 |
| 2–23 months | 321 (49.16) | 807 |
| 2-10 years | 283 (31.83) | 637 |
| 2–5 years | 160 (29,04) | 370 |
| 6–10 years | 123 (36.39) | 267 |
| 11–18 years | 107 (24.83) | 190 |
| 19–34 years | 240 (18.66) | 344 |
| 35–55 years | 188 (28.44) | 284 |
| Solicited local AEs | 838 (21.38) | 1141 |
| 2–23 months | 80 (12.25) | 127 |
| 2–10 years | 267 (30.03) | 461 |
| 2–5 years | 149 (27.04) | 255 |
| 6–10 years | 118 (34.91) | 206 |
| 11–55 years | 491 (20.65) | 553 |
| 11–18 years | 98 (22.74) | 129 |
| 19–34 years | 216 (16.80) | 237 |
| 35–55 years | 177 (26.78) | 187 |
| Solicited systemic AEs | 547 (13.95) | 1121 |
| 2–23 months | 304 (46.55) | 680 |
| 2–10 years | 100 (11.25) | 176 |
| 2–5 years | 67 (12.16) | 115 |
| 6–10 years | 33 (9.76) | 61 |
| 11–55 years | 143 (6.01) | 265 |
| 11–18 years | 29 (6.73) | 61 |
| 19–34 years | 69 (5.37) | 107 |
| 35–55 years | 45 (6.81) | 97 |
| Unsolicited AEs | 186 (4.74) | 268 |
| 2–23 months | 82 (12.56) | 111 |
| 2–10 years | 74 (8.32) | 113 |
| 2–5 years | 56 (10.16) | 87 |
| 6–10 years | 18 (5.33) | 26 |
| 11–55 years | 30 (1.26) | 44 |
| 11–18 years | 8 (1.86) | 12 |
| 19–34 years | 17 (1.32) | 25 |
| 35–55 years | 5 (0.76) | 7 |
| SAEs | 8 (0.20) | 8 |
| 2–23 months | 3 (0.46) | 3 |
| 2–10 years | 3 (0.34) | 3 |
| 2–5 years | 3 (0.54) | 3 |
| 6–10 years | 0 (0.00) | 0 |
| 11–18 years | 2 (0.46) | 2 |
| 19–34 years | 0 (0.00) | 0 |
| 35–55 years | 0 (0.00) | 0 |
| MAAEs ^a^ | 427 (10.89) | 674 |
| 2–23 months | 238 (36.45) | 361 |
| 2–10 years | 148 (16.65) | 249 |
| 2–5 years | 120 (21.78) | 207 |
| 6–10 years | 28 (8.28) | 42 |
| 11–18 years | 20 (4.64) | 31 |
| 19–34 years | 16 (1.24) | 24 |
| 35–55 years | 5 (0.76) | 9 |

AE, adverse event; n (%), number (percentage) of participants with at least one AE; SAE, serious AE; MAEE, medically-attended AE.

Note: ^a^193 cases of MAAEs were also included in solicited systemic (5 cases) and unsolicited (188 cases) AEs.
