# Supplementary material for: Results from a large post-marketing safety surveillance study in the Republic of Korea with a quadrivalent meningococcal CRM-conjugate vaccine in individuals aged 2 months–55 years
Source: Hum Vaccin Immunother. 2019 Oct 25;16(6):1260–7. doi: 10.1080/21645515.2019.1670125 (PMC7482729; doi:10.1080/21645515.2019.1670125)
Supplement: Supplemental Material [file KHVI_A_1670125_SM1774.zip › Yoo et al_HVI_Revised Table S2.docx]

|  | First vaccination (N=304) | | | Second vaccination (N=140) | | | Third vaccination (N=99) | | | Fourth vaccination (N=110) | | |
| --- | --- | --- | --- | --- | --- | --- | --- | --- | --- | --- | --- | --- |
|  | n | % | Number of AEs | n | % | Number of AEs | n | % | Number of AEs | n | % | Number of AEs |
| Solicited AEs |  |  |  |  |  |  |  |  |  |  |  |  |
| Local AE | 34 | 11.18 | 54 | 19 | 13.57 | 31 | 14 | 14.14 | 26 | 13 | 11.82 | 16 |
| Injection site tenderness | 30 | 9.87 | 30 | 15 | 10.71 | 15 | 10 | 10.10 | 10 | 12 | 10.91 | 12 |
| Injection site erythema | 12 | 3.95 | 12 | 9 | 6.43 | 9 | 9 | 9.09 | 9 | 2 | 1.82 | 2 |
| Injection site induration | 12 | 3.95 | 12 | 7 | 5.00 | 7 | 7 | 7.07 | 7 | 2 | 1.82 | 2 |
| Systemic AE | 146 | 48.03 | 353 | 63 | 45.00 | 133 | 46 | 46.46 | 88 | 49 | 44.55 | 106 |
| Change in eating habits | 58 | 19.08 | 58 | 25 | 17.86 | 25 | 13 | 13.13 | 13 | 14 | 12.73 | 14 |
| Sleepiness | 68 | 22.37 | 68 | 20 | 14.29 | 20 | 23 | 23.23 | 23 | 15 | 13.64 | 15 |
| Irritability | 106 | 34.87 | 106 | 49 | 35.00 | 49 | 34 | 34.34 | 34 | 30 | 27.27 | 30 |
| Vomiting | 42 | 13.82 | 42 | 12 | 8.57 | 12 | 8 | 8.08 | 8 | 4 | 3.64 | 4 |
| Diarrhea | 41 | 13.49 | 41 | 13 | 9.29 | 13 | 6 | 6.06 | 6 | 22 | 20.00 | 22 |
| Rash | 7 | 2.30 | 7 | 5 | 3.57 | 5 | 2 | 2.02 | 2 | 6 | 5.45 | 6 |
| Fever | 31 | 10.20 | 31 | 9 | 6.43 | 9 | 2 | 2.02 | 2 | 15 | 13.64 | 15 |
| Unsolicited AEs | 43 | 14.14 | 52 | 16 | 11.43 | 25 | 7 | 7.07 | 11 | 16 | 14.55 | 23 |
| Medically-attended AEs | 113 | 37.17 | 179 | 49 | 35.00 | 74 | 32 | 32.32 | 40 | 44 | 40.00 | 68 |
| Serious AEs | 0 | 0.00 | 0 | 1 | 0.71 | 1 | 1 | 1.01 | 1 | 1 | 0.91 | 1 |
